# Optimized homomorphic encryption solution for secure genome-wide association studies

**DOI:** 10.1186/s12920-020-0719-9

**Published:** 2020-07-21

**Authors:** Marcelo Blatt, Alexander Gusev, Yuriy Polyakov, Kurt Rohloff, Vinod Vaikuntanathan

**Affiliations:** 1 Duality Technologies, Inc., Newark, NJ USA; 2 Dana-Farber Cancer Institute, Boston, MA USA

**Keywords:** Cryptography, Homomorphic encryption, Genome-wide association studies

## Abstract

**Background:**

Genome-Wide Association Studies (GWAS) refer to observational studies of a genome-wide set of genetic variants across many individuals to see if any genetic variants are associated with a certain trait. A typical GWAS analysis of a disease phenotype involves iterative logistic regression of a case/control phenotype on a single-neuclotide polymorphism (SNP) with quantitative covariates. GWAS have been a highly successful approach for identifying genetic-variant associations with many poorly-understood diseases. However, a major limitation of GWAS is the dependence on individual-level genotype/phenotype data and the corresponding privacy concerns.

**Methods:**

We present a solution for secure GWAS using homomorphic encryption (HE) that keeps all individual data encrypted throughout the association study. Our solution is based on an optimized semi-parallel GWAS compute model, a new Residue-Number-System (RNS) variant of the Cheon-Kim-Kim-Song (CKKS) HE scheme, novel techniques to switch between data encodings, and more than a dozen crypto-engineering optimizations.

**Results:**

Our prototype can perform the full GWAS computation for 1,000 individuals, 131,071 SNPs, and 3 covariates in about 10 minutes on a modern server computing node (with 28 cores). Our solution for a smaller dataset was awarded co-first place in iDASH’18 Track 2: “Secure Parallel Genome Wide Association Studies using HE”.

**Conclusions:**

Many of the HE optimizations presented in our paper are general-purpose, and can be used in solving challenging problems with large datasets in other application domains.

## Background

Genome-Wide Association Studies (GWAS) refer to observational studies of a genome-wide set of genetic variants across many individuals to see if any genetic variants are associated with a certain trait. When applied to human data, GWAS typically focus on associations between single-nucleotide polymorhisms (SNPs) and a quantitative or dichotomous disease outcome, as well as a number of quantitative covariates. However, the reliance on full genotype and phenotype data across thousands of samples raises major privacy concerns for GWAS, and has limited their applicability.

Recent work has focused on secure multi-party computation algorithms to facilitate privacy-preserving GWAS, but this approach requires resource-heavy, continuous interactions between users which is impractical for GWAS studies that are aggregated over months or years. To motivate the cryptographic community, the iDASH’18 Organizing Committee ran a special competition track “Secure Parallel Genome Wide Association Studies using Homomorphic Encryption (HE)” to advance the state of the art in GWAS using HE, which is a non-interactive approach to secure computing.

This paper presents our HE-based solution to GWAS. Our solution is based on an optimized GWAS compute model, a new Residue-Number-System (RNS) variant of the Cheon-Kim-Kim-Song (CKKS) HE scheme, novel techniques to switch between data encodings, and more than a dozen crypto-engineering optimizations. The solution can perform the full GWAS computation for 1000 individuals, 131,071 SNPs, and 3 covariates in about 10 minutes on a modern server computing node (with 28 cores).

### Related work

Several other RNS variants of the CKKS HE scheme were independently proposed in 2018. These include the work by Cheon et al. [1], the implementation in Microsoft SEAL 3.0 (released in October 2018), and the variants developed by other teams who submitted their GWAS solutions to the iDASH’18 competition, including UCSD [2] and IBM Research.

## Methods

### Semi-parallel approach of Sikorska et al. [3]

Logistic regression is widely used to model binary response data in GWAS. For instance, it can be used to examine the relationship between disease status (control versus real cases) with respect to phenotypes (age, weight, height, etc.) and genotypes (such as SNP variations). Let *y*_*i*_ denote the disease status for the *i*^th^ individual in a sample of size *N* (*y*_*i*_=1 if the individual is a disease case, and *y*_*i*_=0 otherwise), and $\left (\vec x_{i}^{\prime }, \vec s_{i}\right)$ be the corresponding predictor, where $\vec x_{i}^{\prime } \in \mathbb {R}^{K}$ corresponds to the phenotypes and $\vec s_{i} \in \{0,1,2\}^{M}$ to the genotypes of individual *i* for a set of *K* phenotypes and *M* SNPs. The logistic regression model expresses the relationship between *y*_*i*_ and the predictor set $\left (\vec x_{i}^{\prime }, \vec s_{i}\right)$ in terms of the conditional probability $Pr\left (Y = y_{i}|\vec x_{i}^{\prime }, \vec s_{i}\right)$ of disease, as:

$$Pr\left(y_{i}|\vec x_{i}^{\prime}, \vec s_{i} \right) = \sigma \!\! \left((2y_{i}-1)\left(\boldsymbol{\theta}_{0}^{\prime} + \vec x_{i}^{\prime} \cdot \boldsymbol{\theta}^{\prime} + \vec s_{i} \cdot \boldsymbol{\beta}\right) \right), $$

where *σ* is the logistic function, $\sigma (x) = \frac {1}{1+\exp {(-x)}}$; $\theta _{0}^{\prime } \in \mathbb {R}, \boldsymbol {\theta }^{\prime } \in \mathbb {R}^{K}$ and $\boldsymbol {\beta } \in \mathbb {R}^{M}$ are the *K*+*M*+1 parameters to be determined. For the sake of simplicity, we adopt the canonical notation, that is, $\boldsymbol {\theta } \equiv \left (\theta _{0}^{\prime }, \boldsymbol {\theta }^{\prime } \right) \in \mathbb {R}^{K+1}$ and $\vec x_{i} \equiv \left (1, \vec x_{i}^{\prime }\right) \in \mathbb {R}^{K+1}$ for *i*=1,…,*N*.

Assuming that the effect of each SNP is independent of each other, it is possible to formulate it as a set of *M* independent equations, i.e., decompose the computation into *M* independent logistic regression cases for *K*+1 parameters. Sikorska et al. [3] proposed a “semi-parallel” approach to speed up the logistic regression in the above scenario. The goal is to avoid looping over each SNP by using a vectorized formulation, which includes optimized vector and matrix operations, that allows performing multiple identical actions over different data in a single operation.

The method relies on the assumption that the covariant parameters ***θ*** are nearly the same for all SNPs. This assumption allows the reformulation of fitting *N* vectors in $\mathbb {R}^{K+1}$, followed by a one-step calculation for *M* SNPs at once. Therefore Sikorska’s semi-parallel logistic regression consists of 2 stages:

1. Estimate the coefficients of the clinical covariates, $\boldsymbol {\theta } \in \mathbb {R}^{K+1}$;
2. For each of the *M* SNPs, estimate the corresponding coefficients $\hat {\boldsymbol {\beta }}$ and *p*-value $\vec p \in \mathbb {R}^{M}$.

The first stage, the estimation of $\boldsymbol {\theta }, \hat {\boldsymbol {\theta }}$, was widely addressed in the literature, in particular in the iDASH’17 secure genome analysis competition [4–8].

The second stage, the estimation of the SNP-coefficients $\hat {\boldsymbol {\beta }}$, approximates the optimization problem by a single Newton-Raphson iteration, leading to

$$\hat{\boldsymbol{\beta}} = \mathbf{H}^{-1} \; \mathbf{X}^{\top} \; \mathbf{W} \; \boldsymbol{\zeta}, $$

where **X** is a matrix in $\mathbb {R}^{N \times (K+1)}$ whose rows are the vectors $\vec x_{i}, i = 1, \ldots, N$; $\mathbf {W} \in \mathbb {R}^{N \times N}$ is a diagonal matrix with *ω*_*ii*_=*ρ*_*i*_(1−*ρ*_*i*_) and $\rho _{i} = \sigma \left (\vec x_{i} \cdot \hat \theta ^{(t)}\right)$ for *i*=1,…,*N*; **H**=**X**^⊤^**WX** in $\mathbb {R}^{(K+1) \times (K+1)}$; $\zeta _{i} = \log \left (\frac {\rho _{i}}{1-\rho _{i}}\right) + \frac {y_{i}-\rho _{i}}{\omega _{ii}}, i = 1, \ldots, N$.

Finally, the *z*-value for each parameter *β*_*j*_, for *j*=1,…,*M*, is given by $z_{j} = \frac {\hat {\boldsymbol {\beta }}_{j}}{\epsilon _{j}}$, where $\epsilon _{j} = \sqrt {\left (\mathbf {C}^{-1} \right)_{jj}}$ is the error associated to $\hat {\boldsymbol {\beta }}_{j}$ and **C**=**S**^⊤^**W**(**S**−**XH**^−1^(**X**^⊤^**WS**)). A more compact expression of it is

$$z_{j} = \frac{1}{\det{\mathbf{H}}} \; \frac{\sum_{i}^{n} w_{ii} \zeta_{i}^{*} s_{ij}^{*}}{\sqrt{\sum_{i}^{n} w_{ii} {s_{ij}^{*}}^{2}}} \quad j = 1,\ldots,m,$$

with

$$\begin{aligned} \boldsymbol{\zeta^{*}} = {\det{\mathbf{H}}} \; \boldsymbol{\zeta} - \mathbf{X H^{\dag} X^{\top} W} \; \boldsymbol{\zeta}, \\ \mathbf{S^{*}} = {\det{\mathbf{H}}} \; \mathbf{S} - \mathbf{X H^{\dag} X^{\top} W} \; \mathbf{S}. \end{aligned} $$

where **H**^*†*^ denotes the adjoint of **H**.

### Our approximations

To optimize the efficiency of our HE solution, we introduced several approximations to the semi-parallel method of Sikorska et al. [3].

#### Logistic regression

We found that the gradient descent method is adequate for estimating ***θ***. Starting from an initial ***θ***^(0)^, the gradient descent method at each iteration *t* updates the estimation of the regression parameters

$$ \hat{\boldsymbol{\theta}}^{(t+1)} \leftarrow \hat{ \boldsymbol{\theta}}^{(t)} + \alpha_{t} \mathbf{X} (y + \boldsymbol \rho), $$

where *α*_*t*_ is the learning rate at the *t*-th iteration. Our numerical experiments suggest that a single iteration of the gradient descent procedure with *α*_0_=0.015 and ***θ***^(0)^=0 provides adequate accuracy. For simplicity, we denote *α*_0_ as *α* in the rest of the paper.

#### Logistic function approximation

We used Chebyshev polynomials to approximate the logistic function [9]. From the analysis we performed, we found that a degree-1 approximation *σ*(*x*)=0.5+0.15625*x* provides results with sufficient accuracy. Please refer to “Analysis of our approximations” section for further details.

#### Approximation of ***ζ***

In order to approximate ***ζ***, we considered a Talyor series expansion around $p = \frac {1}{2}$:

$$\begin{aligned} \boldsymbol{\zeta}(p, y) \approx & (-2+4 y) + \\ & (-8+16 y) \left(p-\frac{1}{2}\right)^{2} - \frac{32}{3} \left(p-\frac{1}{2}\right)^{3} + \\ & (-32+64 y) \left(p-\frac{1}{2}\right)^{4} - \frac{256}{5} \left(p-\frac{1}{2}\right)^{5} + \\ & (-128+256 y) \left(p-\frac{1}{2}\right)^{6} - \frac{1536}{7} \left(p-\frac{1}{2}\right)^{7} + \\ & (-512+1024 y) \left(p-\frac{1}{2}\right)^{8}. \end{aligned} $$

#### Matrix inversion and division

Instead of calculating the inverse of the matrix **H**, Cramer’s rule was used: $\mathbf {H}^{-1} = \frac {\text {adj}(\mathbf {H})}{\det (\mathbf {H})}$, where adj(**H**) is the *adjoint* of matrix **H** and det(**H**) is its *determinant*. As the division is an expensive operation, it was deferred to a later stage (after decryption).

#### *p*-value calculation

After computing the *z*-values on the server, the *p*-value computation is performed on the client as depicted in Algorithm 2.

#### Full procedure

The approximations described above were used to create an optimized procedure for the server computation (Algorithm 1). Note that line ?? of Algorithm 1 is the closed form for ***ρ*** that incorporates the parameter estimation of the logistic regression. Therefore $\hat {\boldsymbol {\theta }}$ does not appear explicitly in Algorithm 1.

The annotated encrypted procedure is presented in Algorithm 3. It will be referenced throughout the rest of this section.


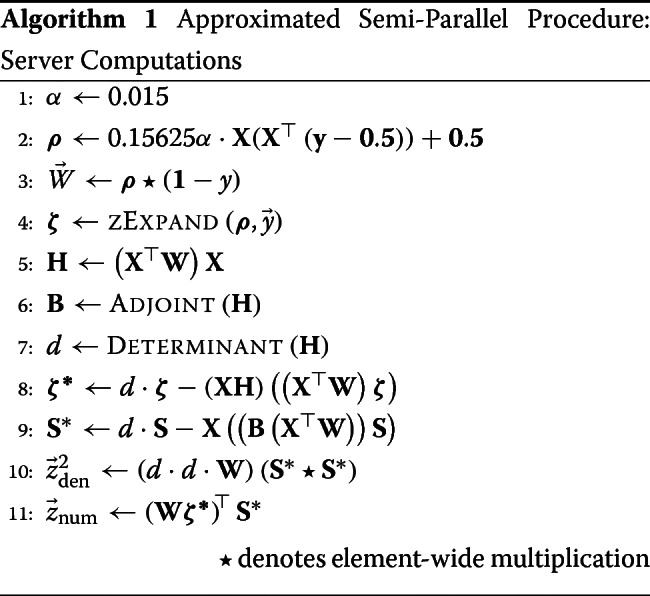



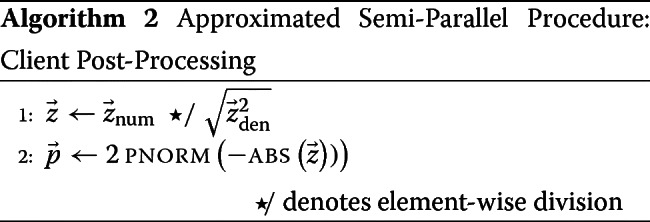


### CKKS scheme

Our solution is based an optimized variant of the Cheon-Kim-Kim-Song scheme [10]. We have developed a Double-Chinese Remainder Theorem (CRT), a.k.a, Residue Number System (RNS), variant of the original scheme. Our variant is based on the same security assumptions as the original scheme, but relies on native 64-bit integer arithmetic instead of multiprecision integer arithmetic for better performance and parallelization.

The original CKKS scheme is formulated for cyclotomic polynomial rings ${\mathcal R} = {\mathbb Z}[x]/\left < x^{n} +1 \right >$, where *n* is a ring dimension that is a power of two (CKKS also supports general cyclotomic rings but they are typically less efficient). The current ciphertext modulus is typically defined as *Q*_*ℓ*_=2^*ℓ*^, i.e., the scheme works with residue rings $\mathcal R_{\ell } = \mathcal R/Q_{\ell } \mathcal R = {\mathbb Z_{2^{\ell }}}[x]/\left < x^{n} +1 \right >$. The algorithms are [10]:

- Setup(1^*λ*^). For an integer *L* that coresponds to the largest ciphertext modulus level, given the security parameter *λ*, output the ring dimension *n*. Set the small distributions *χ*_*key*_,*χ*_*err*_, and *χ*_*enc*_ over $\mathcal R$ for secret, error, and encryption, respectively.
- KeyGen. Sample a secret *s*←*χ*_*key*_, a random *a*→*R*_*L*_, and error *e*←*χ*_*err*_. Set the secret key **sk**←(1,*s*) and public key $\textbf {pk} \leftarrow (b,a) \in \mathcal R^{2}_{L}$, where *b*←−*as*+*e*(mod *Q*_*L*_).
- KSGen_**sk**_(*s*^′^). For $s' \in \mathcal {R}$, sample a random $a' \leftarrow \mathcal {R}_{2 \cdot L}$ and error *e*^′^←*χ*_*err*_. Output the switching key as $\textbf {swk} \leftarrow \left (b', a'\right) \in \mathcal {R}^{2}_{2L}$, where *b*^′^←−*a*^′^*s*^′^+*e*^′^+*Q*_*L*_*s*^′^(mod *Q*_2*L*_). Set **evk**←KSGen_**sk**_(*s*^2^). Set **rk**^(*κ*)^←KSGen_**sk**_(*s*^(*κ*)^).
- Enc_**pk**_(*m*). For $m \in \mathcal {R}$, sample *v*←*χ*_*enc*_ and *e*_0_,*e*_1_←*χ*_*err*_. Output **ct**←*v*·**pk**+(*m*+*e*_0_,*e*_1_)(mod *Q*_*L*_).
- Dec_**sk**_(**ct**). For $\textbf {ct} = (c_{0},c_{1}) \in \mathcal {R}_{\ell }^{2}$, output $\tilde {m} = c_{0} + c_{1} \cdot s \left (\bmod Q_{\ell } \right)$.
- CAdd(**ct**,*c*). For $\textbf {ct} = (b, a) \in \mathcal {R}_{\ell }^{2}$ and $c \in \mathcal {R}$, output **ct**_cadd_←(*b*+*c*,*a*)(mod *Q*_*ℓ*_).
- Add(**ct**_1_,**ct**_2_). For $\textbf {ct}_{1},\textbf {ct}_{2} \in \mathcal {R}_{\ell }^{2}$, output **ct**_add_←**ct**_1_+**ct**_2_(mod *Q*_*ℓ*_).
- CMult(**ct**,*c*). For $\textbf {ct} \in \mathcal {R}_{\ell }^{2}$ and $c \in \mathcal {R}$, output **ct**_cmult_←*c*·**ct**(mod *Q*_*ℓ*_).
- Mult_**evk**_(**ct**_1_,**ct**_2_). For $\textbf {ct}_{i} = (b_{i},a_{i}) \in \mathcal {R}_{\ell }^{2}$, let (*d*_0_,*d*_1_,*d*_2_)=(*b*_1_*b*_2_,*a*_1_*b*_2_+*a*_2_*b*_1_,*a*_1_*a*_2_)(mod *Q*_*ℓ*_). Output $\textbf {ct}_{\text {mult}} \leftarrow (d_{0}, d_{1}) + \lfloor Q_{L}^{-1} \cdot d_{2} \cdot \textbf {evk} \rceil \left (\bmod Q_{\ell } \right)$.
- $\textsc {Rotate}_{\textbf {rk}^{(\kappa)}}(\textbf {ct},\kappa)$. For $\textbf {ct} = (b,a) \in \mathcal {R}_{\ell }^{2}$ and rotation index *κ*, output $\textbf {ct}_{\text {rotate}} \leftarrow \left (b^{(\kappa)}, 0\right) + \lfloor Q_{L}^{-1} \cdot a^{(\kappa)} \cdot \textbf {rk}^{(\kappa)} \rceil \left (\bmod Q_{\ell } \right)$.
- ReScale (**ct**, *p*). For a ciphertext $\textbf {ct} \in \mathcal {R}_{\ell }^{2}$ and an integer *p*, output **ct**^′^←⌊2^−*p*^·**ct**⌉(mod (*Q*_*ℓ*_/2^*p*^)).

The CKKS scheme supports an efficient packing of *r* (up to *n*/2) real numbers into a single ciphertext. The encoding and decoding operations are defined as follows:

- Encode (**w**, *p*). For $w \in \mathbb {R}^{r}$, output the polynomial $m \leftarrow \lfloor \phi (2^{p} \cdot \mathbf {w}) \rceil \in \mathcal {R}$.
- Decode (*m*, *p*). For a plaintext $m \in \mathcal {R}$, output the polynomial $\mathbf {w} \leftarrow \phi ^{-1}\left (m/2^{p}\right) \in \mathbb {R}^{r}$.

Here, *ϕ*(*x*) is a certain complex canonical embedding map, which is similar conceptually to inverse Fourier transform.

### Our RNS variant of the CKKS scheme

Our CKKS variant performs all operations in RNS. In other words, the power-of-two modulus *Q*_*ℓ*_=2^*ℓ*^ is replaced with $\prod _{i=1}^{\ell } q_{i}$, where *q*_*i*_ are same-size prime moduli satisfying *q*_*i*_≡1 mod 2*n* (for efficient number theoretic transforms (NTT) that convert native-integer polynomials w.r.t. each CRT modulus from coefficient representation to the evaluation one, and vice versa). The primes are chosen to be as close to 2^*p*^ as possible to minimize the error introduced by rescaling.

The two major changes in our variant compared to the original CKKS scheme deal with rescaling and key switching. We also made two other minor changes. First, we use the ternary random discrete distribution for *χ*_*key*_ and *χ*_*enc*_ instead of the sparse distributions as the lattice attacks for this case are better studied, and the ternary distribution is included in the HE standard [11]. Second, we do additional scaling of plaintexts and ciphertexts to support the use of RNS (only native integer arithmetic) during encoding/decoding.

#### Rescaling in RNS

To efficiently perform rescaling in RNS from *Q*_*ℓ*_ to *Q*_*ℓ*−1_, we replace the scaling down by 2^*p*^ with scaling down by *q*_*ℓ*_. We choose all *q*_*i*_, where *i*∈[*L*], such that 2^*p*^/*q*_*i*_ is in the range (1−2^−*ε*^,1+2^−*ε*^), where *ε* is kept as small as possible. To minimize the cumulative approximation error growth in deeper computations, we also alternate *q*_*i*_ w.r.t. 2^*p*^. For instance, if *q*_1_<2^*p*^, then *q*_2_>2^*p*^ and *q*_3_<2^*p*^, etc.

The new rescaling operation to scale down by one level is defined as

- ReScaleRNS (**ct**). For a ciphertext $\textbf {ct} \in \mathcal {R}_{\ell }^{2}$, output $\textbf {ct}' \leftarrow \lfloor q_{\ell }^{-1} \cdot \textbf {ct} \rceil \left (\bmod Q_{\ell -1} \right)$.

We derive the procedure for computing $ \lfloor q_{\ell }^{-1} \cdot \textbf {ct} \rceil \left (\bmod Q_{\ell -1} \right)$ using the CRT scaling technique proposed in [12]. Consider the following CRT representation of a multiprecision integer $x \in \mathbb {Z}_{Q_{\ell }}$:

1 $$ x = \sum_{i=1}^{\ell} x_{i} \cdot {\tilde{q}_{i}} \cdot {q_{i}^{*}} -\upsilon' \cdot \ Q_{\ell} ~\text{for some}~\upsilon' \in\mathbb{Z},  $$

where

$$ {q_{i}^{*}} = Q_{\ell}/q_{i} \in \mathbb{Z} ~\text{and}~ {\tilde{q}_{i}} = {q_{i}^{*}}^{-1} \pmod{q_{i}} \in \mathbb{Z}_{q_{i}}. $$

Then we can write

$$\frac{x}{q_{\ell}} = \frac{1}{q_{\ell}}\left(\sum\limits_{i=1}^{\ell-1} x_{i} \tilde{q}_{i} q^{*}_{i} + x_{\ell}\tilde{q}_{\ell} q^{*}_{\ell} - \upsilon' Q_{\ell}\right). $$

After rounding and applying the modulo reduction, the last term is removed yielding

2 $$\begin{array}{@{}rcl@{}} \left\lfloor \frac{x}{q_{\ell}} \right\rceil \equiv \sum\limits_{i=1}^{\ell-1} x_{i} \cdot \frac{\tilde{q}_{i} q^{*}_{i}} {q_{\ell}} + \left\lfloor x_{\ell}\cdot \frac{\tilde{q}_{\ell} q^{*}_{\ell}}{q_{\ell}} \right\rceil \left(\bmod Q_{\ell-1} \right). \end{array} $$

The first term can be directly computed in RNS by summing up the products of *x*_*i*_ and $q_{\ell }^{-1} \left (\bmod q_{i}\right)$. For the second term, we precompute the residues of $\left \lfloor \frac {\tilde {q}_{\ell } q^{*}_{\ell }}{q_{\ell }} \right \rfloor $ and multiply them by the corresponding residues of *x*_*ℓ*_ during rescaling. Then we add the fractional part, which has the residue of ⌊*x*_*ℓ*_/*q*_*ℓ*_⌉, i.e., 0 or 1, for each CRT modulus *q*_*i*_. Note that the fractional part is negligibly small and hence can be excluded from the implementation.

The computational complexity of rescaling is determined by the computation in the second term of (2). We first need to run one native inverse NTT for residues w.r.t. *q*_*ℓ*_ and then *ℓ*−1 native NTTs to go back to the evaluation representation. All the computations in the first term of (2) are done directly in evaluation representation. Therefore, each rescaling operation requires *ℓ* native-integer NTTs.

The maximum approximation error introduced by rescaling from *ℓ* to *ℓ*−1 is $\left \vert q_{\ell }^{-1} \cdot m - 2^{-p} \cdot m \right \vert \le 2^{-\epsilon } \cdot \left \vert 2^{-p} \cdot m \right \vert $.

This procedure can be easily generalized to support scaling down by multiple CRT moduli. This case is similar to the first stage of complex scaling in CRT representation described in Section 2.4 of [12].

#### Key switching

For key switching, we use the CRT decomposition key switching algorithm that was originally proposed in [13] and improved in [12] for the Brakerski/Fan-Vercauteren (BFV) scheme. The advantages of this technique vs. the one used in the original CKKS scheme (initially proposed for the Brakerski-Gentry-Vaikuntanathan scheme in [14]) are that this technique has lower computational complexity for relatively small numbers of levels (up to 8 or so), and does not require an approximately two-fold increase in the ring dimension to support the appropriate lattice security level. Both of these benefits were important for our solution.

The operations of the CKKS scheme that are modified by the key switching procedure are rewritten as:

- KSGenRNS_**sk**_(*s*^′^). For $s' \in \mathcal {R}$, sample a random $a_{i}' \leftarrow \mathcal {R}_{L}$ and error *ei*′←*χ*_*err*_. Output the switching key as $\textbf {swk} \leftarrow \left \{ \left (b'_{i}, a'_{i}\right)\right \}_{i \in [L]} \in \mathcal {R}^{2 \times L}_{L}$, where $b^{\prime }_{i} \leftarrow -a'_{i} s' + e'_{i} + {\tilde {q}_{i}} \cdot {q_{i}^{*}} \cdot s' \left (\bmod Q_{L} \right)$. Set **evk**←KSGenRNS_**sk**_(*s*^2^). Set **rk**^(*κ*)^←KSGenRNS_**sk**_(*s*^(*κ*)^).
- MultRNS_**evk**_(**ct**_1_,**ct**_2_). For $\textbf {ct}_{i} = (b_{i},a_{i}) \in \mathcal {R}_{\ell }^{2}$, let (*d*_0_,*d*_1_,*d*_2_)=(*b*_1_*b*_2_,*a*_1_*b*_2_+*a*_2_*b*_1_,*a*_1_*a*_2_)(mod *Q*_*ℓ*_). Decompose *d*_2_ into its CRT components $\phantom {\dot {i}\!}[d_{2}]_{q_{i}}$ and output
  $$\textbf{ct}_{\text{mult}} \leftarrow (d_{0}, d_{1}) + \sum\limits_{i=1}^{\ell} [d_{2}]_{q_{i}} \cdot \textbf{evk}_{i} \left(\bmod Q_{\ell} \right). $$
- $\textsc {RotateRNS}_{\textbf {rk}^{(\kappa)}}(\textbf {ct},\kappa)$. For $\textbf {ct} = (b,a) \in \mathcal {R}_{\ell }^{2}$, output
  $$\textbf{ct}_{\text{rotate}} \leftarrow \left(b^{(\kappa)}, 0\right) + \sum\limits_{i=1}^{\ell} \left[a^{(\kappa)}\right]_{q_{i}} \cdot \textbf{rk}^{(\kappa)}_{i} \left(\bmod Q_{\ell} \right), $$
  where $\left [a^{(\kappa)}\right ]_{q_{i}}$ are CRT components of *a*^(*κ*)^.

Each key-switching operation requires one inverse NTT (*ℓ* native-integer NTTs) to switch *d*_2_ (or *a*^(*κ*)^ for rotation) from evaluation to coefficient representation and then *ℓ* NTTs (*ℓ*^2^−*ℓ* native-integer NTTs) to go back to evaluation representation for each CRT component. Hence, the total complexity in terms of native-integer NTTs is *ℓ*^2^.

This key switching procedure also supports a second level of decomposition by extracting base-*w* digits in each residue using the procedure described in Appendix B.1 of [13].

#### Noise estimates

We present here heuristic noise estimates for the RNS variant of CKKS using the canonical embedding norm, which corresponds to the infinity norm for the evaluation of a polynomial $\mathcal {R}$ at 2*n* complex roots of unity. For more details on the canonical embedding mapping and norm, the reader is referred to [10]. The main differences between our expressions and those in [10] are due to the use of ternary uniform distribution and a different key switching technique.

- **Encoding and Encryption.** The bound for fresh encryption $B_{\text {clean}}=6 \sigma \left (4 \sqrt {3} n + \sqrt {n} \right)$, where *σ* is the standard deviation for error distribution. The decoding is correct as long as 2^*p*^>*n*+2*B*_clean_.
- **Addition.** The bound for homomorphic addition *B*_add_=*B*_1_+*B*_2_, where *B*_*i*_ is the noise bound for *i*-th ciphertext.
- **Rescaling.** The noise bound for rescaling is $B_{\text {rescale}} = q_{\ell }^{-1} \cdot B + B_{\text {scale}}$, where *B* is the input noise and $B_{\text {scale}}=\sqrt {3} \left (12 n + \sqrt {n} \right)$.
- **Rotation.** The noise bound for rotation (key switching) is $B_{\text {ksw}}=\frac {8}{\sqrt {3}} \cdot n \sigma w \left \lceil \log _{w} {q_{\ell }} \right \rceil $.
- **Multiplication.** If we have two ciphertexts **ct**_1_ and **ct**_2_ with $\left \lVert m_{1} \right \rVert ^{\text {can}}_{\infty } < \nu _{1}$, noise bound *B*_1_ and $\left \lVert m_{2} \right \rVert ^{\text {can}}_{\infty } < \nu _{2}$, noise bound *B*_2_, respectively, the noise bound *B*_mult_=*ν*_1_*B*_2_+*ν*_2_*B*_1_+*B*_1_*B*_2_+*B*_ksw_.

In most cases, the parameter selection is determined by the multiplicative depth and the approximation error in rescaling. The approximation error (with about *ε* bits being “erased” by rescaling) dominates the noise growth of other operations and should be done last (after a multiplication). The only practical exception is when rotations are performed before any multiplications. In this case, the key switching noise may be high if the *w*-base is large, e.g., comparable to 2^*p*^ as in the case of CRT decomposition without further digit decomposition of each residue.

#### Comparison to the RNS variant by Cheon et al. [1]

Both our RNS variant of CKKS and the variant proposed by Cheon et al. [1] work with an RNS basis consisting of native-integer primes *q*_*i*_ that are close to 2^*p*^ (with *ε* bits of precision). In other words, scaling down by 2^*p*^ is replaced with approximate scaling down by *q*_*ℓ*_. Hence the rescaling approach in both variants is similar. The techniques for the scaling operation itself are different, but the computational complexity of both scaling techniques appears to be the same (requiring *ℓ* native-integer NTTs).

The key switching-procedure developed in [1] is based on the approach originally proposed for the Brakerski-Gentry-Vaikuntanathan scheme [14], which requires doubling the ciphertext modulus (and roughly doubling the ring dimension). We use the residue/digit decomposition approach originally proposed in [13] and improved in [12]. Our key-switching technique requires more NTTs but provides better overall performance for relatively “shallow” circuits (our estimates suggest this approach should be faster up to 8 levels or so).

### Plaintext encoding

Our solution uses two kinds of plaintext encoding. Initially, **X** and **y** are packed in single ciphertexts similar to how it was done in [8]. We denote this as *packed-matrix encoding*. All matrix products in steps 2 through 8 of Algorithm 3 use the rotation-based SUMROWVEC and SUMCOLVEC procedures from [5]. Later in the algorithm (starting from step 9), the solution switches to single-integer ciphertexts for **X** and the vectors and matrices derived from **X** and **y**. We call the latter encoding as *packed-integer encoding*. As a result of this, our matrix operations with the SNPs data (first appearing in step 9) involve only cheap SIMD multiplications and additions of packed-integer and packed-row-vector ciphertexts, and do not involve any expensive rotations. All operations before computing on the SNPs data are performed using packed-matrix (single) ciphertexts.

#### Packed-matrix encoding

The packed-matrix encoding packs a full matrix or vector into a single ciphertext, cloning as many entries as needed to support matrix-matrix and matrix-vector products. The cloning makes it possible to minimize the number of computationally expensive rotations in matrix-matrix (vector) products.

We encode/encrypt both **X** and **X**^⊤^ to avoid calling transposition in the encrypted domain. We pack $\mathbf {X} \in \mathbb {R}^{N \times k}$ in a row-wise order, cloning each row *k*−1 times before going to the next row. Here, we introduce *k*=*K*+1 for brevity.

$$\mathbf{X} =\left[\begin{array}{cccc} X_{11} & X_{12} & \dots & X_{1k} \\ X_{11} & X_{12} & \dots & X_{1k} \\ \vdots & \vdots & \vdots & \vdots \\ X_{21} & X_{22} & \dots & X_{2k} \\ X_{21} & X_{22} & \dots & X_{2k} \\ \vdots & \vdots & \vdots & \vdots \\ X_{N1} & X_{N2} & \dots & X_{Nk} \\ X_{N1} & X_{N2} & \dots & X_{Nk} \\ \end{array}\right] $$

We pack $\mathbf {X}^{\top } \in \mathbb {R}^{k \times N}$ by taking each element of matrix **X** (marshalling it in the row-wise order) and cloning it to form a complete row.

$$\mathbf{X}^{\top} = \left[\begin{array}{cccc} X_{11} & X_{11} & \dots & X_{11} \\ X_{12} & X_{12} & \dots & X_{12} \\ \vdots & \vdots & \vdots & \vdots \\ X_{1k} & X_{1k} & \dots & X_{1k} \\ \vdots & \vdots & \vdots & \vdots \\ X_{N1} & X_{N1} & \dots & X_{N1} \\ X_{N2} & X_{N2} & \dots & X_{N2} \\ \vdots & \vdots & \vdots & \vdots \\ X_{Nk} & X_{Nk} & \dots & X_{Nk} \\ \end{array}\right] $$

Both matrices require *N*·*k*^2^ slots.

We pack $\mathbf {y} \in \mathbb {R}^{N}$ column-wise by cloning **y***k*^2^−1 times to the right. That is, we have

$$\mathbf{y} = \underbrace{ \left[\begin{array}{cccc} y_{1} & y_{1} & \dots & y_{1} \\ y_{2} & y_{2} & \dots & y_{2} \\ \vdots & \vdots & \vdots & \vdots \\ y_{N} & y_{N} & \dots & y_{N} \\ \end{array}\right] }_{k^{2} \,\, \text{cloned values}} $$

The resulting vector ***ρ*** is represented the same way as **y**. Both use *N*·*k*^2^ slots.

The diagonal matrix **W** is represented as a vector by extracting the diagonal, and the resulting vector is packed in the same format as ***ρ***.

The SNPs matrix **S** is encoded either as an array of ciphertexts (when *M*>*n*/2) or a single ciphertext (when *M*≤*n*/2) without any cloning, i.e., the classical SIMD packing of vectors is used.

Matrices and vectors, such as **X** and **y**, can be encoded in a single ciphertext as long as *N*·*k*^2^≤*n*/2. If this condition does not hold, the packing can be trivially extended to multiple ciphertexts per matrix/vector.

#### Packed-integer encoding

To support efficient matrix multiplication without rotations, we also encode **X** as *N*·*k* single-integer ciphertexts. In this case, each entry of **X** is cloned to all slots of a single ciphertext. We denote such packing of **X** as **X**_1_.


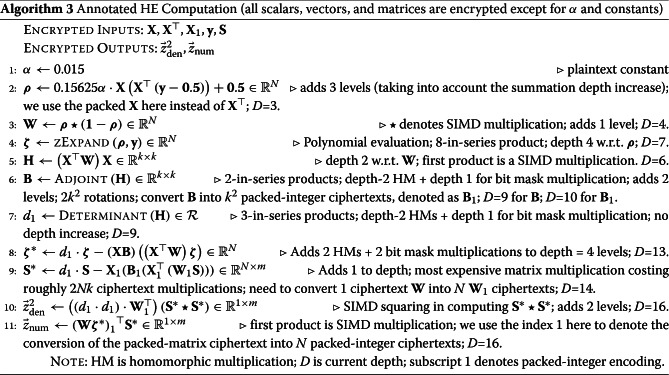


### Conversion from packed-matrix to packed-integer encoding

The main bottleneck of our solution is the conversion of vectors from a packed-matrix ciphertext to multiple packed-integer ciphertexts. We have developed and implemented three different methods for performing this conversion. Based on the requirements for performance and scalability, we chose one of these methods for our prototype.

To illustrate the problem and its solutions, we consider the task of converting the packed-matrix single-ciphertext encryption of **y** into *N* packed-integer ciphertexts. A similar task has to be executed twice in our algorithm for secure GWAS.

#### Method 1: *N*⌈log*n*⌉ rotations

Our first solution can be summarized as follows:

1. Fill all *n*/2 slots of **y** by cloning existing *N*·*k*^2^ slots. This requires $\log \left (n/\left (2 \bar {N} \cdot k^{2} \right) \right)$ rotations and additions. The cloning procedure is described in [8]. Here, $\bar {N} = 2^{\lceil \log N\rceil }$.
2. Run *N* bit mask multiplications to form *N* ciphertexts each containing $n/\left (2 \bar {N}\right)$ cloned values for each component of **y**. All other slots are zeroed out.
3. Clone existing $n/\left (2 \bar {N}\right)$ non-zero values to all slots in each of the *N* ciphertexts. This operation requires *N*⌈log*N*⌉ rotations and additions, and is the main bottleneck of the computation.

#### Method 2: $\bar {N}$ rotations and ⌈log*N*⌉ depth increase

The idea of our second solution is to represent the conversion as a binary tree. At each level *i* of the tree we perform *i* rotations, 4·*i* bit mask multiplications, and 2·*i* additions, getting two output ciphertexts from each input ciphertext. Although this recursive method requires only $\bar {N}$ rotations, 4$\bar {N}$ bit mask multiplications, and 2$\bar {N}$ additions, there is a ⌈log*N*⌉ depth increase due to bit mask multiplications at each level of the binary tree.

To illustrate this approach, consider a simpler case (the logic would stay the same when we clone *y*_*i*_ any number of times):

$$\left[ y_{1} y_{2} y_{3} \cdots y_{N-2} y_{N-1} y_{N}\right]. $$

First rotate by -1 and get

$$Rot_{1}(y) = \left[ y_{N} y_{1} y_{2} \cdots y_{N-3} y_{N-2} y_{N-1}\right]. $$

Then multiply both *y* and *Rot*_1_(*y*) by *M*_1_=[101010⋯10] and *M*_2_=[010101⋯01], and sum up two possible combinations, yielding

$$y_{1,1} = y \star M_{1} + Rot_{1}(y) \star M_{2} = \left[ y_{1} y_{1} y_{3} y_{3} \cdots y_{N-1} y_{N-1}\right], $$

$$y_{1,2} = y \star M_{2} + Rot_{1}(y) \star M_{1} = \left[ y_{N} y_{2} y_{2} \cdots y_{N-2} y_{N-2} y_{N}\right]. $$

Next compute *Rot*_2_(*y*_1,1_) and *Rot*_2_(*y*_1,2_), multiply *y*_1,1_ and *y*_1,2_ and their rotations by [110011⋯1100] and [001100⋯0011] for each pair, and sum up four possible combinations. Now there are 4 *y*_2,*i*_ items.

We recursively execute this procedure until the end.

#### Method 3: $\bar {N}^{2}$ bit mask multiplications and $\bar {N}$ rotations

Another approach achieving *N* rotations can be summarized as follows:

1. Fill all *n*/2 slots of **y** by cloning existing *N*·*k*^2^ slots.
2. Compute $\bar {N}-1$ cheap rotations of the original ciphertext using the hoisting procedure from [15].
3. For each component of **y**, do $\bar {N}$ bit mask multiplications (one per rotation) that would extract the component and zero out all other slots.
4. For each component of **y**, do $\bar {N}-1$ additions of masked ciphertexts.

Although this procedure requires only roughly $\bar {N}$ cheap rotations, it involves $\bar {N}^{2}$ bit mask multiplications and additions, which now become the main bottleneck for relatively large values of *N*.

#### Comparison of the methods

We implemented all three methods, and carried out both complexity and practical performance comparison.

As *N* is relatively large (at least 245), $\bar {N}^{2}$ bit mask multiplications in Method 3 resulted in computation runtimes that are at least 2x-3x larger than Method 1 with *N*⌈log*N*⌉ rotations. However, Method 3 would be faster for smaller *N*, e.g., less than 100.

Method 2 is a good option only when the depth increase can be incorporated in the existing circuit without increasing the overall circuit depth. But the scalability of this approach is questionable. The depth increase of ⌈log*N*⌉ = 8 could not be integrated in the circuit of our solution, and thus we chose Method 1 for our implementation.

Note that in our implementation the depth cost of bit mask multiplication is the same as for homomorphic multiplication, which implies there is room for improvement. Therefore, a more depth-efficient bit mask multiplication procedure may result in a significantly better performance for Method 2, possibly superior to that of Method 1.

### Minimizing the number of key switching operations

One of the optimization goals for our solution is to reduce the number of key switching operations, which are used both for rotation and relinearization (after homomorphic multiplication). Each such operation has a high computational complexity, i.e., requires *ℓ*^2^ native-integer NTTs. We have optimized our algorithm to minimize the number of key switching operations. For instance, all computations involving encrypted SNPs data require only 16 (*k*^2^) key switching operations in total. A great majority of the computations involving encrypted SNPs data use only “cheap” SIMD multiplications and additions, and sparingly rescaling operations.

#### Multiplications with lazy or no relinearization

In steps 9 through 11 of Algorithm 3, our procedure calls only 16 (*k*^2^) relinearizations. In other words, all large-dimension SIMD products are performed without relinearization (the ciphertext size is allowed to grow). The procedure calls the relinearization procedure only when multiplying by **B**_1_ in step 9, which works with the smallest dimension (*k*) in the chained matrix product. We refer to this deferred relinearization as “lazy” relinearization. Any homomorphic multiplications after this product are performed without a single relinearization, which significantly reduces the runtime of computation.

#### Use of additions instead of rotations

The packed-integer encoding is introduced in steps 9 through 11 of Algorithm 3 to replace any rotation-based summations over rows/columns with SIMD homomorphic additions. The only places where the rotations are used are to homomorphically convert **B**,**W**, and (**W*ζ***^∗^) from packed-matrix encoding to the packed-integer one. The use of rotation-based summation in the chained product of step 9 would require a substantially larger number of rotations as compared to the conversion of two vectors of size *N* and one matrix of size *k*×*k*.

### Minimizing the number of NTTs

Besides key switching, NTTs are used for rescaling. In some cases, expensive rotations can be replaced with hoisted automorphisms from [15], reducing the number of NTTs for multiple rotations of the same ciphertext to the NTT cost of a single rotation. Our solution minimizes the number of rescaling operations and uses hoisted automorphisms where applicable.

#### Use rescaling sparingly

We use the following techniques to minimize the number of rescaling operations:

- When there are homomorphic multiplications followed by aggregation of ciphertexts, such as addition of multiple ciphertexts, we apply rescaling after the aggregation, i.e., we call it once rather than for every homomorphic multiplication.
- If there is a benefit in lazy rescaling, e.g., when the number of ciphertexts at the following level is much smaller, we defer rescaling until later. In this case, we have to make sure the depth requirement is not increased, which is true when one of the multiplicands is scaled w.r.t. 2^*p*^ rather a power of it.
- The rescaling operations are not called at the end of computation if skipping them does not increase the multiplicative depth of the circuit.

#### Hoisted automorphisms

Hoisted automorphisms are useful when multiple rotations of the same ciphertext need to be computed [15]. Our solution encounters this scenario when computing the matrix inversion of **H** in steps 6 and 7 of Algorithm 3, and hence the hoisted automorphisms are used there in favor of regular rotations.

### Minimizing the noise growth and ciphertext modulus

We minimized the noise growth/ciphertext modulus of the computation circuit using the following techniques:

- Binary tree multiplication was employed for any chained products of ciphertexts.
- Closed-form expressions (such as in step 2 of Algorithm 3) were derived to get the maximum benefit from binary tree multiplication.
- Binary tree addition for any summation of a large number of ciphertexts was employed to achieve a *O*(log*N*) noise growth.
- To guarantee that the end result of the computation requires only one native-integer polynomials, we multiplied both numerator and denominator by estimated scaling factors (different from 2^*p*^). These factors were introduced during bit mask multiplications to avoid any extra depth increase due to this additional scaling.
- The maintenance operations of HE, such as key switching and rescaling, were properly ordered to minimize the noise growth. For instance, rescaling was done after the rotations following a multiplication (not before).

### Harnessing the CRT ladder

As the circuit evaluation progresses, the number of CRT limbs, i.e., native polynomials in the Double-CRT structure, gets reduced due to rescaling. For instance, at level *ℓ* the number of CRT limbs is reduced by *L*−*ℓ* as compared to fresh ciphertexts. This provides a speedup in CKKS compared to scale-invariant schemes, such as BFV. We can further take advantage of the decreasing CRT “ladder” by encrypting plaintexts at the level they are first used and by compressing evaluation keys as the computation progresses. This reduces storage requirements. We also minimize the number of CRT limbs by finding the minimum number of limbs needed for correct result (starting from the end of the computation circuit). Below we provide some examples of how these techniques are applied in our solution.

#### Encrypt ciphertexts at the level first used

As the SNPs matrix **S** is first used in step 9 of Algorithm 3 (after 10 levels of computation), we encrypt it using 7 CRT limbs rather than 17 corresponding to the initial ciphertext modulus. This reduces the storage requirements for the SNPs matrix by a factor of 2.4x.

#### Compress evaluation keys as needed

Same rotation keys are used multiple times throughout the computation. Whenever they are no longer required below a certain level, we compress them to the current level, thus reducing the number of CRT limbs. Note that the rotation keys consume most of the space utilized by public keys in our solution.

#### Use the lowest number of CRT limbs for ciphertexts

Once the lowest multiplicative depth for the circuit is determined, we choose the actual level for ciphertexts by counting from the end of the circuit (not from the beginning) up to the specific computation. This minimizes the number of CRT limbs used, thus reducing both runtime and storage requirements.

Consider the example of **S**. If we were to count the level from the beginning of the circuit, we would choose level 8 (to match the level of **B**_1_). But we choose 10 instead because the maximum depth of computations from **S** in step 9 to the end of the circuit is 6. This gives more than 1.5x runtime improvement for the rotations in the conversion from **W** to **W**_1_, which is done immediately before computing **W**_1_**S**. The storage requirement for **S** is also reduced by roughly a factor of 1.3x.

### Matrix inversion

As pointed out earlier, we use Cramer’s rule to compute the matrix inverse of **H**. The numerator is the adjoint of **H** while the denominator is the determinant of **H**. To extract specific components of **H**, we use cheap rotations (hoisted automorphisms) followed by bit mask multiplications to clear out the values that are not used. As both numerator and denominator contain a lot of common products of the rotations for **H**, we wrote both of them down in the closed form and compute common products only once. The closed form for the determinant also allows the direct application of binary tree multiplication (3-in-series products require a binary depth of 2). The depth cost of these steps is 3 (2 for homomorphic multiplications and 1 for bit mask multiplication).

When computing the determinant and *k*^2^ components in the adjoint, all homomorphic multiplications are performed without relinearization, and the relinearization is applied at the very end (for each component) after all additions and subtractions are done. This significantly reduces the number of expensive key switching operations when computing the matrix adjoint and determinant.

The procedure for computing the adjoint and determinat also prepares the packed-matrix variant of **B** for computing ***ζ***^∗^ in step 8 and the packed-integer variant **B**, i.e., **B**_1_, for computing **S**^∗^ in step 9 by performing appropriate rotations and additions. The final rescaling for the components in the adjoint and determinant is done after all rotations are computed. Otherwise the noise growth in rotations would lead to incorrect results after decryption.

### Order of products in matrix chain multiplication

The order of matrix products in matrix chain multiplications has a major effect on the performance of our solution. The two most complex and costly chained matrix products in Algorithm 3 are step 8 (computation of ***ζ***^∗^) and step 9 (computation of **S**^∗^). Typically the matrix chain multiplication problem is an optimization problem that can be solved using dynamic programming. In the case of regular plaintext computations, the goal is usually to minimize the number of element multiplications. In the encrypted solution, additional constraints are introduced, and these constraints can be different depending on the plaintext encoding used, as illustrated below.

In step 8, we work with a chain of single ciphertexts (packed matrix encoding). The constraints for this case can be summarized as follows:

- Make sure the outcome of each intermediate product is a single ciphertext. For instance, we cannot have a product where outer dimensions are both *N*.
- The costs of SumRowVec and SumColVec are different. The latter requires a bit mask multiplication, and the number of rotations corresponds either to row or column size. The possible constraints are to minimize the number of rotations and/or minimize the depth of bit mask multiplications.
- Minimize the depth of the overall circuit. In other words, the term at highest level should be given special attention. The binary tree multiplication technique should also be properly applied.

In step 9, we work with products of many packed-integer ciphertexts and *N* SIMD-packed ciphertexts (for each row of matrix **S**). The guidelines for optimization in this case can be summarized as follows:

- Minimize the total number of SIMD multiplications.
- Minimize the depth of the overall circuit. In other words, the term at highest level should be given special attention. The binary tree multiplication technique should also be properly applied.

In our solution, the decisions regarding the order of matrix chain multiplication were done by hand. But in a more general case, where the computation circuit is built automatically, one would have to include algorithms for finding the optimal order by solving the appropriate dynamic optimization problem.

### Loop parallelization

To benefit from multi-core CPU environments, our solution applies loop parallelization at various levels.

At the encryption stage, the parallelization is done for the loop iterating over all individuals (size *N*, which is at least 245). This implies the encryption runtime should decrease almost linearly with the number of physical cores.

In the computation stage, the following loop parallelizations are applied:

- All matrix products in $\mathbf {X}_{1} \left (\mathbf {B}_{1} \left (\mathbf {X}_{1}^{\top } \, \left (\mathbf {W}_{1} \mathbf {S}\right)\right)\right)$ at step 9 of Algorithm 3 are parallelized over inner dimensions (*N* or *k*, depending on the product).
- All SIMD products in steps 10 and 11 of Algorithm 3 are parallelized over *N*.
- In matrix inversion, the extraction of *k*^2^ components of **H** is parallelized over *k*^2^.
- In the homomorphic encoding conversion routine of Method 1, the parallelization is applied to the main loop over *N*.
- Loop parallelization is also applied in many places at the level of CKKS and lower-lever ring operations. In the case of NTTs for polynomials in Double-CRT representation, the parallelization is done over *ℓ*. In the case of RNS subroutines, the parallelization is applied at the level of polynomial coefficients (dimension *n*).

## Results

### Dataset

Our experiments were performed using the training dataset provided by the iDASH 2018 organizers. The training data were extracted from the Personal Genome Project (https://www.personalgenomes.org/us). The dataset includes 245 individuals, 10,643 SNPs, and 3 covariates. We also generated larger datasets for scalability analysis by re-sampling the original dataset.

### Software implementation

We implemented our solution in PALISADE v1.2 [16]. We added our own implementation for the RNS variant of the CKKS scheme to PALISADE. For loop parallelization, we used OpenMP.

### Parameter selection

The parameters used are summarized below. According to [11], our parameters correspond to at least 128 bits of security for classical computers.

- The size of ciphertext modulus *Q*_*L*_ for fresh ciphertexts is 850 bits.
- The ring dimension *n* is 2^15^=32,768.
- The number of CRT limbs in the fresh ciphertext modulus is 17 (*L*=17), which corresponds to 16 levels in the computation circuit. Each CRT modulus is 50 bits long.
- Number of bits *p* in the plaintext scaling factor of CKKS scheme is 50. For this value of *p*, the approximation error introduced by each rescaling typically affected up to 25 least significant bits of the encrypted data.
- The key switching window matches the size of CRT moduli, i.e., 50 bits.
- We use the ternary secret key distribution, i.e., random integers between -1 and 1, as commonly done for BFV.
- The error distribution parameter *σ* is 3.19.

### Performance results

#### Storage requirements

The maximum (initial) storage requirements for the case of *N*=245; *M*=10,643; *K*=3 are summarized in Table 1. The storage requirements take into account that **S** and **X**_1_ are first used at *ℓ*=7 and *ℓ*=6, respectively. The rotation key size is computed as a sum of space requirements for 16 keys at *ℓ*=17, 13 at *ℓ*=12, and 12 at *ℓ*=9. The relinearization keys are used from the start of the computation (*ℓ*=17). The sizes of public and secret keys are relatively small: 4.7 and 8.5 MB, respectively.

**Table 1:** Maximum storage requirements for *N*=245; *M*=10,643; *K*=3

Ciphertexts [GB]					Evaluation Keys [GB]	
**X**	**X**^⊤^	**y**	**S**	**X**_1_	Rotation	Relinearization
0.0085	0.0085	0.0085	0.84	2.87	3.65	0.42

The encryption storage requirements in practical settings can be reduced by converting homomorphically the encrypted packed-matrix ciphertext **X** to *N* packed-integer ciphertexts, i.e., **X**_1_, on demand. This can be done as an offline operation, resulting in an approximately 4x reduction in fresh ciphertext size.

#### Execution time and peak memory utilization

Table 2 reports the runtimes and peak RAM utilization observed for the official iDASH evaluation environment and a 28-core server node. The results suggest that it takes about 3.5 minutes and about 10 GB of RAM (all ciphertexts and keys are stored in memory) to evaluate homomorphically the GWAS procedure for 245 individuals, 14,841 SNPs, and 3 covariates on a 4-core Amazon instance. The runtime and storage requirements for the case of 1,000 individuals, 131,071 SNPs, and 3 covariates for a modern server computing node (2 x 14 cores) are about 10 minutes and 116 GB, respectively.

**Table 2:** Runtimes and peak RAM utilization on a UTHealth ITS VM (4 cores, 16 GB RAM, 200 GB hard drive, AWS T2 Xlarge equivalent, official iDASH’18 evaluation environment) and a server node with 2 x 14 cores of Intel(R) Xeon(R) CPU E5-2680 v4 at 2.40GHz (500 GB RAM and 2 TB hard drive)

System	*N*	*M*	KeyGen	Enc	Eval	Dec	Peak RAM
			[min]	[min]	[min]	[s]	[GB]
UTHealth ITS VM (iDASH)	245	14,841	0.35	0.34	3.46	0.06	9.99
28-core server node	245	10,643	0.12	0.059	1.45	0.06	12.2
28-core server node	300	20,000	0.12	0.088	1.88	0.11	16.2
28-core server node	1,000	131,071	0.12	0.72	10.44	0.4	116

#### Accuracy analysis

**Fig. 1:**
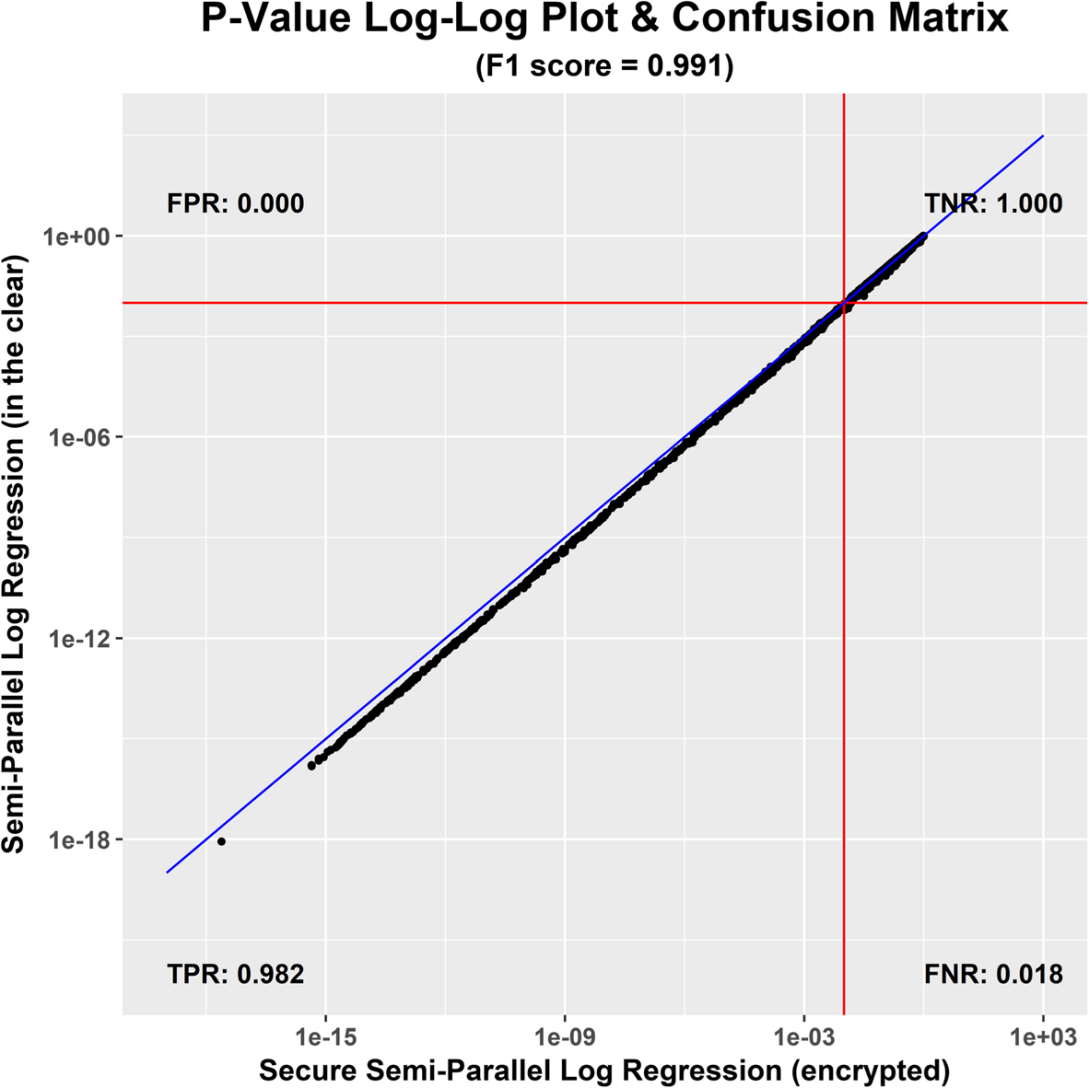
Accuracy of our encrypted computing prototype w.r.t the plaintext reference implementation [3]

We compared the accuracy of the *p*-values computed using our HE prototype with a plaintext reference implementation of the semi-parallel method proposed by Sikorska et al. [3]. The results for the case of *N*=245 and *M*=10,643 are summarized in Fig. 1. The graphs visualize the confusion table when choosing 0.01 as a threshold to classify SNPs as significant or not (depicted as the red lines). It is a log-log plot of the *p*-values obtained by the two different approaches. The vertical axes correspond to the semi-parallel logistic regression and horizontal axes to the *p*-values obtained by the HE computation. The diagonal blue line depicts the case when the two classifiers provide exactly the same *p*-value for each input data.

Each quadrant corresponds to one of possible outcomes: true positive (both classify a SNP as significant), false positive (the semi-parallel model as not significant and the HE computation as significant), true negative (both classify a SNP as significant) and false negative (the semi-parallel model as significant and the HE computation as not significant). The graph shows the true positive rate (TPR), false positive rate (FPR), true negative rate (TPR) and false negative rate (FNR). We use F1 score as a single index to summarize the performance. The graph suggests that the error introduced by our approximation is negligibly small (F1 score of 0.991).

#### Analysis of our approximations

As described in “Our approximations” section, there are 3 compute-model parameters that affect the approximation error: the highest degree of the Chebyshev polynomials used to approximate the logit-function, *d*_*l*_; the degree the Taylor expansion of *ζ*,*d*_*z*_; and the number of iterations, *t*, for the gradient descent procedure. Clearly, there is a trade-off between the accuracy of the approximation and the depth of the computation circuit, which determines the computational complexity.

In order to avoid over-fitting, we also used other data sets from the Harvard Personal Genome Project [17]. We ran experiments for different conditions reported in the PGP Participant Survey and found that the approximation of *ζ* has a significant impact on the quality of the results, and is highly sensitive to the choice of cases and disease populations. Therefore, we selected a relatively high degree for the Taylor expansion, *d*_*z*_=8, that provides adequate accuracy for unbalanced populations of up to 10%/90%. Note that the data used for the iDASH competition was relatively balanced.

We found that increasing the number of iterations, *t*, and the degree *d*_*l*_ of the Chebyshev polynomials used to approximate the logit-function has a relatively minor effect on the accuracy of our solution. As an example, Table 3 shows that the *F*_1_ score for the *p*-value with threshold 0.01 does not significantly change with increase in *d*_*l*_, while the expected computational cost of using a higher depth would be substantial.

**Table 3:** *F*_1_ score as a function of the degree *d*_*l*_ of the Chebyshev polynomials used to approximate the logit-function at *d*_*z*_=8 and *t*=1

*d*_*l*_	1	3	5	7	9	11	13	15
*F*_1_	0.9914	0.9924	0.9927	0.9931	0.9932	0.9933	0.9933	0.9933

#### Profiling

Table 4 reports the breakdown of runtimes for three different cases. The results for *N*=245,*M*=10,643 suggest that the conversion of vectors from the packed-matrix to packed-integer encoding is the bottleneck for the single-threaded case. However, the conversion procedure parallelizes better (improving by a factor of 15.3x on a 28-core machine) than most of the other operations, effectively reducing its contribution from 77% in the single-threaded experiment to 38% for the 28-threaded experiment. The experiments for larger numbers of SNPs imply that the contribution of the conversion procedure further declines as its computational complexity does not depend on *M*.

**Table 4:** Runtime profiling on the 28-core node; time in seconds; numbers in header row denote step #’s in Algorithm 3; numbers in parentheses are for the single-threaded experiment; → denotes the conversion from packed-matrix to packed-integer encoding

*N*	*M*	1–5	6–7 + **B**→**B**_1_	8	**W**→**W**_1_	9	10	**W*ζ***^∗^→(**W*ζ***^∗^)_1_	11
245	10,643	13.3	23.4	4.6	27.4	10.4	1.8	5.5	0.62
		(27.4)	(40.2)	(6.5)	(419)	(59.3)	(12.0)	(84.1)	(1.64)
300	20,000	13.1	23.5	4.6	33.2	25.7	3.8	7.3	1.5
1,000	131,071	12.7	22.9	4.2	132.8	360.6	47.2	25.0	21.0

As the maximum size of individuals did not exceed 1,024 in our experiments, all operations in Steps 1–8 of Algorithm 3 worked with single ciphertexts, and the runtime of these steps stayed approximately the same for all experiments. At the same time, the contribution of the matrix products involving **S** (steps 9 through 11) significantly increased (from 15% for *N*=245,*M*=10,643 to 68% for *N*=1,000, *M* = 131,071).

## Discussion

The solution presented in this work was awarded first place (along with another solution from UCSD) in the iDASH’18 competition (Track 2: Secure Parallel Genome Wide Association Studies using Homomorphic Encryption). Hence it represents the state of the art in secure GWAS using homomorphic encryption.

The main limitations of our solution are (1) the need to know the computation and parameters of the semi-parallel procedure in advance and (2) the hand-tuned nature of many optimizations applied to our solution. The first problem can be solved once the bootstrapping for the CKKS scheme becomes more practical. The second challenge can be tackled once automated compilers for homomorphic encryption are developed. Both are open research problems.

## Conclusions

The results demonstrate that our solution is able to perform the full GWAS computation homomorphically for 1000 individuals, 131,071 SNPs, and 3 covariates in about 10 minutes on a modern server computing node. Many of the optimizations presented in our paper are general-purpose and can be applied to solving challenging problems dealing with large datasets in other application domains. The major general-purpose optimizations include a new RNS variant of the CKKS scheme and multiple methods of homomorphic switching between data encodings.

## Data Availability

The iDASH 2018 competition data was only available to registered competition participants.
